# Supplementary material for: From sequence to enzyme mechanism using multi-label machine learning
Source: BMC Bioinformatics. 2014 May 19;15:150. doi: 10.1186/1471-2105-15-150 (PMC4229970; doi:10.1186/1471-2105-15-150)
Supplement: Additional file 2 — Java code of ml2db. Additional file ml2db_code.tar.gz contains the Java source code to run the multi-label machine learning experiments and save the results to database. The code’s Javadoc is included. [file 1471-2105-15-150-S2.zip › additional file 2/ml2db/ml2db/doc/main/ml2db/al/utility/pickers/byfrequency/ByFrequencyPicker.html]

ByFrequencyPicker


JavaScript is disabled on your browser.


- Overview
- Package
- Class
- Use
- Tree
- Deprecated
- Index
- Help

- Prev Class
- Next Class

- Frames
- No Frames

- All Classes

- Summary:
- Nested |
- Field |
- Constr |
- Method

- Detail:
- Field |
- Constr |
- Method


main.ml2db.al.utility.pickers.byfrequency

## Class ByFrequencyPicker

- java.lang.Object
- - main.ml2db.al.utility.UtilityMetric
  - - main.ml2db.al.utility.pickers.RandomPicker
    - - main.ml2db.al.utility.pickers.byfrequency.ByFrequencyPicker

- All Implemented Interfaces:
  :   Picker

  Direct Known Subclasses:
  :   AttributesByFrequencyPicker, AttSetByFrequencyPicker, LabelsByFreqPicker, NearestNeighboursByFreqUtility

  ---

    

  ```
  public abstract class ByFrequencyPicker
  extends RandomPicker
  ```

  Picks each of the features (class, attribute or attribute set) by descending
  order of frequency, and then, uniformly randomly, one of the instances of
  that feature. Then it starts over again until it has exausted all the
  instances.

  Version:
  :   3 May 2012

  Author:
  :   Luna De Ferrari ldf(at)st-andrews.ac.uk or
      luna.deferrari@st-andrews.ac.uk

- - ### Field Summary

    - ### Fields inherited from class main.ml2db.al.utility.pickers.RandomPicker

      `m_debug, m_test`
  - ### Constructor Summary

    Constructors

    Constructor and Description

    `ByFrequencyPicker()`
  - ### Method Summary

    Methods

    | Modifier and Type | Method and Description |
    | --- | --- |
    | `void` | `calculateFrequency()` Orders the attribute set by descending frequency |
    | `java.util.Vector<java.lang.String>` | `getFeaturesByDescendentFrequency()` |
    | `uk.ac.standrews.utils.main.maputils.OneToManyMap<java.lang.Integer,java.lang.String>` | `getFrequencyToFeatureMap()` |
    | `abstract java.lang.String` | `getMetricName()` |
    | `void` | `initialise()` |
    | `void` | `orderAttsetByDescFreq()` |
    | `void` | `setFeaturesByDescendentFrequency()` |
    | `void` | `setFrequencyToFeatureMap()` |

    - ### Methods inherited from class main.ml2db.al.utility.pickers.RandomPicker

      `getInstanceToFeaturesMap, getQueries, getQuery, getStep, getUtility, isDebug, isTest, refresh, setDebug, setFeatureToInstanceMap, setStep, setTest`
    - ### Methods inherited from class main.ml2db.al.utility.UtilityMetric

      `getUnlabelled, setUnlabelled`
    - ### Methods inherited from class java.lang.Object

      `equals, getClass, hashCode, notify, notifyAll, toString, wait, wait, wait`

- - ### Constructor Detail


    - #### ByFrequencyPicker

      ```
      public ByFrequencyPicker()
      ```
  - ### Method Detail


    - #### calculateFrequency

      ```
      public void calculateFrequency()
      ```

      Orders the attribute set by descending frequency

      Parameters:
      :   `attributeSetInstanceMap` -


    - #### getFeaturesByDescendentFrequency

      ```
      public java.util.Vector<java.lang.String> getFeaturesByDescendentFrequency()
      ```

      Returns:
      :   m\_featuresByDescendentFrequency


    - #### getFrequencyToFeatureMap

      ```
      public uk.ac.standrews.utils.main.maputils.OneToManyMap<java.lang.Integer,java.lang.String> getFrequencyToFeatureMap()
      ```

      Returns:
      :   m\_frequencyToFeatureMap


    - #### getMetricName

      ```
      public abstract java.lang.String getMetricName()
      ```

      **Specified by:**
      :   `getMetricName` in class `RandomPicker`


    - #### initialise

      ```
      public void initialise()
      ```

      **Specified by:**
      :   `initialise` in class `UtilityMetric`


    - #### orderAttsetByDescFreq

      ```
      public void orderAttsetByDescFreq()
      ```


    - #### setFeaturesByDescendentFrequency

      ```
      public void setFeaturesByDescendentFrequency()
      ```

      Parameters:
      :   `featuresByDescendentFrequency` - m\_featuresByDescendentFrequency to set


    - #### setFrequencyToFeatureMap

      ```
      public void setFrequencyToFeatureMap()
      ```

      Parameters:
      :   `frequencyToFeatureMap` - m\_frequencyToFeatureMap to set


- Overview
- Package
- Class
- Use
- Tree
- Deprecated
- Index
- Help

- Prev Class
- Next Class

- Frames
- No Frames

- All Classes

- Summary:
- Nested |
- Field |
- Constr |
- Method

- Detail:
- Field |
- Constr |
- Method
